# One Solution for Two Challenges: The Lizard *Microlophus atacamensis* Avoids Overheating by Foraging in Intertidal Shores

**DOI:** 10.1371/journal.pone.0097735

**Published:** 2014-05-19

**Authors:** Maritza Sepúlveda, Pablo Sabat, Warren P. Porter, José Miguel Fariña

**Affiliations:** 1 Centro de Investigación y Gestión en Recursos Naturales (CIGREN), Instituto de Biología, Facultad de Ciencias, Universidad de Valparaíso, Valparaíso, Chile; 2 Departamento de Ciencias Ecológicas, Facultad de Ciencias, Universidad de Chile, Santiago, Chile; 3 Department of Zoology, University of Wisconsin, Madison, Wisconsin, United States of America; 4 Departamento de Ecología, Facultad de Ciencias Biológicas, Pontificia Universidad Católica de Chile, Santiago, Chile; 5 Department of Ecology and Evolutionary Biology, Brown University, Providence, Rhode Island, United States of America; Pennsylvania State University, United States of America

## Abstract

In lizards, one of the most important behavioral mechanisms to cope with spatial and temporal variations in thermal resources observed is activity time. The longer a lizard can maintain activity, the more time it has to forage and reach larger adult body size. We studied the behavioral adjustments to different climatic regimens on daily and seasonal scales in three natural populations of the lizard *Microlophus atacamensis* along a latitudinal temperature and rainfall gradient. We also used Niche Mapper to determinate the amount of thermally suitable time for activity for this species. Abundance and daily activity patterns varied greatly over the year for the three populations. In summer and spring, the daily activity times were greater, and were reduced in fall and winter seasons. In summer, when stressful heat loads should prohibit activity over a midday gap, lizards did not show bimodal patterns of activity. Instead, they move to the cooler intertidal habitat. Abundance and thermal quality in the southernmost coolest site was lower, and the potential annual activity time decreases with latitude. Contrary to expectations, lizards from this locality showed the largest body sizes possibly due to diet and/or time to sexual maturation. Our results indicate that the intertidal habitat is a key factor that influences daily and seasonal activity of *M. atacamensis* lizards. While this habitat is not climatically optimal for lizards, it allows them to behaviorally extend their activity window and gain access to food in the intertidal areas.

## Introduction

One of the principal challenges for animals is to obtain enough food to provide nutrients and energy needed for maintenance, survival, growth, and reproduction [1]. The energy allocated to these processes ultimately depends on the daily and annual energy budget, which in turn depends on activity time [2]. In lizards, it has been demonstrated that the daily and seasonal activity times may be influenced by both endogenous and exogenous environmental factors [3], [4]. The endogenous rhythms of activity are driven both by circadian and circannual clocks that allow changes in the duration of daily activity across seasons, allowing the lizard to anticipate the upcoming season [5]. However, environmental exogenous factors may mask endogenous rhythms by either increasing or suppressing activity, the most important being ambient temperatures in the field, which influence both daily and seasonal activity patterns [3], [6], [7], [8]. Assuming that lizards are foraging while active, and that food is not limiting in a given environment, changes in daily or seasonal activity times due to spatial and temporal variations in thermal resources influence the daily prey capture rate [2], [9], [10], thus ultimately affecting energy acquisition and the reproductive success of lizards.

Daily and seasonal activity may also lead to specific geographic patterns in growth and body size. Lizards with longer activity seasons are expected to grow faster [2]. For example, in sagebrush lizards, it was found that longer seasonal activity was associated with an increase in individual growth and larger adult body size [11] (but see [2]). Therefore, variation in thermal environment, through its effect on thermoregulatory behavior and activity time, may have a profound and direct impact on time budgets of the lizards, and cause some of the observed variation in life histories among widespread populations [2], [9], [12], [13].

The family *Tropiduridae* is mostly composed by terrestrial feeders, but some species that dwell in desert coastal areas (with low levels of terrestrial productivity), feed almost exclusively on intertidal zones [14], [15]. In Chile, two species from the genus *Microlophus* use this feeding strategy: *Microlophus quadrivittatus* and *M. atacamensis* [16]. The geographical range of *M. quadrivitattus* (from Arica 18°00′S to Antofagasta 20°32′S) falls into an area where the extreme desert reaches the coast and there is practically no annual precipitation (maximum recorded  = 2 mm year^−1^) [17], whereas the geographical range of *M. atacamensis*, the subject of this study and southernmost species of the genus, runs 500 km into the so-called Desert Coastal area which presents a gradient of precipitation from 2 mm year^−1^ in the North (at Antofagasta) to almost 120 mm year^−1^ in the South (La Serena 29°55′S) [18]. For this species it is reported that the diet of this species is composed both of intertidal (algae and crustacea) and terrestrial (isopoda, coleoptera) prey [18]. The importance of each of these dietary items is associated with spatial and temporal variation in the productivity levels of both habitats, with an increasing proportion of terrestrial items in a north to south gradient [18], [19].

The intertidal zone is an unfavorable thermal environment because lizards lose heat quickly by conduction, convection and evaporation when feeding on a wet rock substrate [20]. For that reason, lizards may face a trade-off between feeding and thermoregulation. Because *M. atacamensis* populations are under different thermal constraints at latitudinal, seasonal and microhabitat scales, a temporal and geographical variability in their thermal biology is expected. Lack of physiological intraspecific variation in thermal biology in *M. atacamensis* (e.g. selected body temperatures and heating and cooling rates) suggests that lizards may compensate adequately the variations in their thermal environment through a behavioral mechanism instead of physiological adjustment [21].

Through field observations and using a process-driven mechanistic model (Niche Mapper) [22] we explored the behavioral adjustments of *M. atacamensis* to different climatic regimens, and their potential for activity at different temporal and spatial scales. We selected three populations along the entire geographical range of this species. Considering that environmental temperature decreases as latitude increases, we specifically assessed whether daily and seasonal activity patterns are modified with increasing latitude. Associated with this hypothesis, it is expected that body size should decrease in populations that exhibit shorter activity times.

## Materials and Methods

### Organism and study sites

From July 2005 to April 2006 we studied the abundance and activity patterns in three sites on the northern Chilean coast: Medano (24°37′S; 70°33′W), Zenteno (26°51′S; 70°49′W), and Arrayán (29°41′S; 71°19′W). Climatological data for the study sites are presented in Table 1. Medano and Zenteno are under a coastal desert climatic regime, characterized by less than 2 mm of rain per year [17]. The southernmost locality, Arrayán, in contrast, falls into the per-arid Mediterranean zone. Rainfall in this site averages 120 mm/year. Terrestrial productivity also shows a gradient, with a significant increment of plant cover, from ca. 0% in Medano to 23% in Arrayán [18]. Lizards live along the intertidal zone in a stretch of approximately 150–200 m dominated by flat areas of a mixture of rocks (with heights from 0.2 to 3 m), pebbles, cobblestones, and sand [23].

**Table 1:** Climatological data for the study sites in the four seasons of the year.

Season		Locality		
		Medano	Zenteno	Arrayán
Summer	T_max_	22.9±0.8	23.2±1.7	20.0±1.8
	T_min_	18.6±1.0	16.9±1.2	15.8±0.9
	T_mean_	20.7±1.7	19.6±2.5	17.6±1.8
Fall	T_max_	19.7±2.3	20.0±2.3	16.9±2.4
	T_min_	15.6±2.3	14.0±2.4	12.7±2.4
	T_mean_	17.6±2.7	16.8±2.9	14.5±2.6
Winter	T_max_	16.5±1.1	17.9±1.5	15.4±1.5
	T_min_	13.2±1.1	11.9±2.1	11.1±1.4
	T_mean_	14.9±1.5	14.6±2.4	13.0±1.9
Spring	T_max_	18.9±1.6	19.9±1.9	17.4±1.6
	T_min_	16.0±1.3	14.1±2.4	13.3±1.3
	T_mean_	17.5±1.8	16.7±2.3	15.0±1.9
Relative humidity (%)		85.6	-	84.5
Precipitation (mm)		2.2	2.2	127.4
Climate		Desert	Desert	Mediterranean

*Note*: Values are expressed as mean ± SD. Data were obtained from the Servicio Hidrológico y Oceanográfico of the Armada de Chile. Only precipitation was obtained from [17].

### Abundance

For each site, and every four months, the abundance of active *M. atacamensis* lizards at intertidal and terrestrial areas was assessed by visual counts. On each occasion, and for two consecutive days, censuses data were collected by walking parallel to the shoreline, covering both intertidal and the adjacent terrestrial (supralittoral) substrates. Censuses were made at 0900, 1100, 1300, 1500, and 1700 h, walking on each occasion for one hour and covering approximately 1 km of coast. The maximum number of lizards sighted during a census period was used as a relative index of abundance. This index does not provide a measure of the actual density of lizards, but allows the between-habitats and between-seasons comparison of abundance at a geographical scale [24]. Lizards were differentiated by age class (juveniles or adults), recording their position on intertidal or terrestrial habitats. Air and soil temperatures (T_air_ and T_soil_) were measured immediately prior and after each census at the beginning and end of the transect.

### Daily activity patterns

Simultaneously with the abundance analysis, one observer counted lizards from 0900 to 1800 h in a 1500 m^2^ area, recording the number of lizards sighted every 30 min. We selected this time window because *M. atacamensis* is a diurnal lizard that restricts their activity to daytime hours when T_soil_ is above 22°C [18]. As above, lizards were differentiated according to age classes (juveniles or adults) and substrate (intertidal or terrestrial habitat). To establish an approximation of thermal conditions of the habitat occupied by lizards, we placed six Tidbit temperature data loggers with an internal temperature sensor (three in the intertidal zone and three in terrestrial zones) at randomly selected microsites for each season and locality. Temperatures in the data loggers were registered every 30 min. Thermal profiles collected from the electronic devices do not differ substantially from copper lizards models exposed to the same conditions, suggesting that Tidbit could be used directly to monitor operative temperatures (T_e_) [25]. To test if this assumption was true for our model species, in the laboratory we calibrated the tidbits measurements with those of dead lizards placed in a thermal gradient. The results showed that data recorded from electronic devices are highly correlated with temperatures from dead *M. atacamensis* lizards exposed to identical thermal conditions (*r* = 0.99).We also calculated the *d_e_* index, which represents the thermal quality of the habitat, from the lizard's perspective [26]. This index is calculated as the mean of the absolute value of the deviations of T_e_ from selected temperature (T_sel_) of the corresponding site and season. It was found that *M. atacamensis* lizards have a T_sel_ of 34.3°C, independent of localities or seasons [21]. For this reason, we use this value for thermal quality measurements.

### Activity times

To estimate the maximum duration of daily and annual activity of lizards from the three sites, we used the Niche Mapper model [6], [22], to determinate the biophysical effects on the amount of thermally suitable time for activity, under realistic climatic conditions [27]. The microclimate model in Niche Mapper allows the translation from macro to microclimatic environmental variables relevant to the organisms. More details of these models can be found in [6], [22], and at [http://www.zoology.wisc.edu/faculty/Por/Por.html].

The microclimate model contains a subroutine for computing solar radiation given a specific date, time, latitude, longitude, slope, and aspect [28]. Time dependent input data consist of monthly 2 m shade air temperatures, wind speed, and relative humidities. These data for the three sites were obtained from the Servicio Hidrológico y Oceanográfico of the Armada de Chile (Table 1). The microclimate model calculates the hourly intensity of solar radiation and the profiles of T_air_ and T_soil_, wind and humidity above the surface. These data were used for calculating the hottest (maximum sunlight available) and coolest (maximum shade available) microclimates available to the lizards in the rocks for each hour of the day and for each location. Hourly output from the microclimate model for the hottest and coolest above and below ground conditions were used to define the full range of environmental conditions available locally. Time dependent input data used for the microclimate simulations can be found in Tables 1, 2 and 3. The microclimate output conditions were then used as input for the ectotherm model, in addition to various properties of the animal relating to morphological, physical, physiological, food, and behavioral properties [27].

**Table 2:** Parameters used in Niche Mapper for *Microlophus atacamensis* lizards.

Variable	Value	Source
Mean male/female weight, Medano (g)	65.2/35.7	This study
Mean male/female weight, Zenteno (g)	81.2/35.8	This study
Mean male/female weight, Arrayán (g)	84.9/45.8	This study
Oxygen extraction efficiency (%)	6–12	Assumed
Activity above basal multiplier	3	Assumed
Preferred body temperature, C	33.7–34.7	[21]
Voluntary thermal minimum, C	28	[21]
Voluntary thermal maximum, C	37	[21]
Fecal water (%)	10	Assumed
Dry matter of food (%)	31	Assumed
Dry mass of food carbohydrate (%)	12	Assumed
Dry mass of food lipids (%)	18	Assumed
Dry mass of food protein (%)	67	Assumed
Activity period	Diurnal	This study
Percent skin wet	0	Assumed
Maximum/minimum absorptivity (%)	92/85	ASD measurements. This study

**Table 3:** Mean ± SE of air, soil, and operative temperature.

Season	Air temperature (T_air_, °C)			Soil temperature (T_soil_,°C)			Operative temperature (T_e_,°C)		
	M	Z	A	M	Z	A	M	Z	A
Summer	20.4±0.5	24.4±0.6	22.3±0.1	31.2±1.5	36.8±1.5	33.4±2.2	33.3±1.0	37.6±1.1	37.5±1.2
Fall	20.0±0.5	20.4±1.9	18.4±1.0	30.9±2.5	26.5±0.2	23.6±3.4	30.0±1.2	25.4±1.1	20.6±0.9
Winter	18.3±0.2	15.6±0.7	18.2±1.4	24.8±1.3	18.2±1.2	20.1±2.8	20.9±0.8	23.1±0.9	21.9±1.0
Spring	18.0±0.0	13.7±0.5	18.7±1.3	33.8±0.4	23.5±1.7	26.3±1.0	28.9±1.3	22.5±0.7	27.8±1.3

Data are based on two sampling days of each season. M: Medano, Z: Zenteno, A: Arrayán.

The ectotherm program uses the results from the microclimate model to determine which hours of the day an ectotherm can stay within its preferred body temperature range. We used this model to examine the frequency distribution of expected daytime body temperature (T_b_) of *M. atacamensis* lizards throughout the year in the three sites. The model assumes that the animal is always active (and exposed to local wind and humidity) when thermal conditions permit. If conditions are too hot or cold for activity, the animal retreats (close to the sea or underground) to a temperature as close to its optimal T_b_ as possible. Analyses were done separately for wet and dry conditions, simulating both intertidal and terrestrial substrates, respectively. Calculations for the wet substrates assumed a 100% wet surface. Wet and dry reflectivities of local substrates were measured using an ASD portable spectroreflectometer for the energy balance calculations for all habitats. The spectral range of these measurements was 350–2500 nm, approximately 97% of the sunlight reaching the surface of the earth. Rock reflectivities were 21/32% (dark/light) for Medano, 21% for Zenteno, and 16% for Arrayán. Input data for the ectotherm simulations can be found in Table 2.

### Body size

Live lizards from the three populations were captured during the study period (N = 55 for Medano, N = 50 for Zenteno, and N = 27 for Arrayán). Two measures of body size were registered at each capture: (1) snout-vent length (SVL), which was measured to the nearest millimeter using a caliper, and (2) mass, which was measured to the nearest 0.1 g using a Pesola scale. The analyses were restricted to adults and, because this species exhibits a clear sexual dimorphism in body size [16], [23], sexes were analyzed separately. As soon as the measurements were taken the individuals were liberated.

### Ethics statement

The protocol of capture was done with research permits from the Chilean Agriculture and Livestock Bureau (SAG) (Permit Number 98), and under the approval of the Pontificia Universidad Católica de Santiago Ethics Committee. No specific permissions were required in the study sites because they are within an open access zone of the Atacama Desert coast. Also, *M. atacamensis* is classified as Vulnerable under the Chilean Hunting Law (DS 5).

### Statistical analyses

The abundance of *M. atacamensis* was analyzed with a two-way ANOVA [29], using sites and seasons as factors, with 3 and 4 levels, respectively. Kendall-rank correlations were used to determine whether the mean number of lizards was correlated with T_air_ or T_soil_. Means of T_air_ and T_soil_ were used to compute this statistic. Thermal conditions during the daily activity patterns (using the *d_e_* index) were compared among sites and seasons using a two-way ANOVA. Body size data (SVL and mass) was analyzed with a two-way ANOVA, using sites and sex as factors. Normality of the data was checked by Shapiro-Wilk's test of normality and, when necessary, the data were log-transformed. Post-hoc comparisons were performed using a Tukey's honest significance difference test. All comparisons were performed using Statistica 8.0 for Windows [30].

## Results

### Abundance

**Figure 1:**
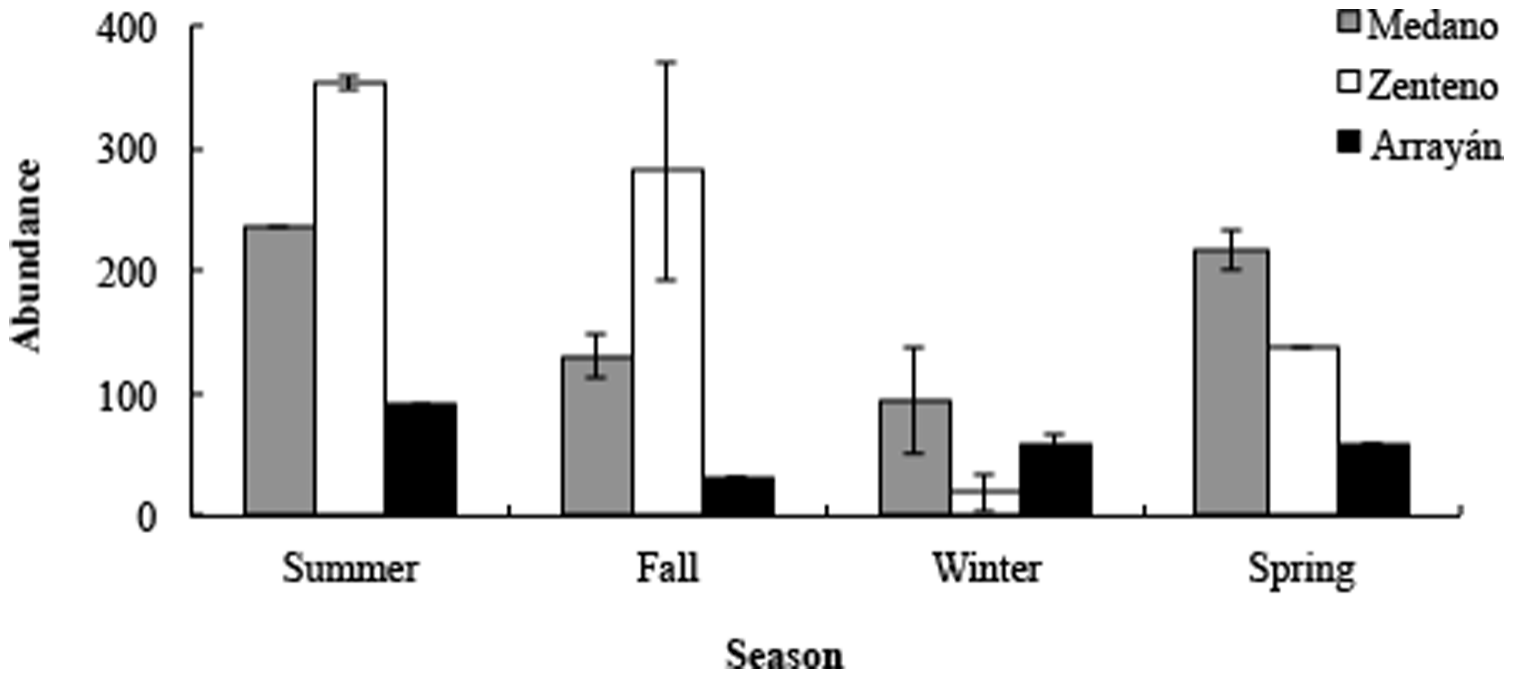
Seasonal and geographical comparisons of mean lizard abundance. Vertical lines represent standard errors.

The abundance of *M. atacamensis* was significantly different among sites (*F*_2,12_ = 14.40, *P*<0.001), seasons (*F*_3,12_ = 9.73, *P* = 0.002), and for the interactions between these factors (*F*_6,12_ = 4.44, *P* = 0.014). Highest mean values occurred in Medano and Zenteno (at the north and mid geographical range, respectively) compared to the southernmost site (Arrayán) (Fig. 1). In terms of seasonality, fewer individuals were found in winter, compared to the summer. T_air_, T_soil_, and T_e_ were higher in summer, related to the other seasons (Table 3).T_air_ and T_soil_ were significantly correlated with the number of lizards observed during the censuses, for the three sites studied (τ = 0.49 and τ = 0.35 in Medano; τ = 0.35 and τ = 0.40 in Zenteno; τ = 0.18 and τ = 0.14 in Arrayán, for T_air_ and T_soil_, respectively) (all Kendall rank correlation coefficients significant at *P*<0.001).

### Daily and annual activity patterns

**Figure 2:**
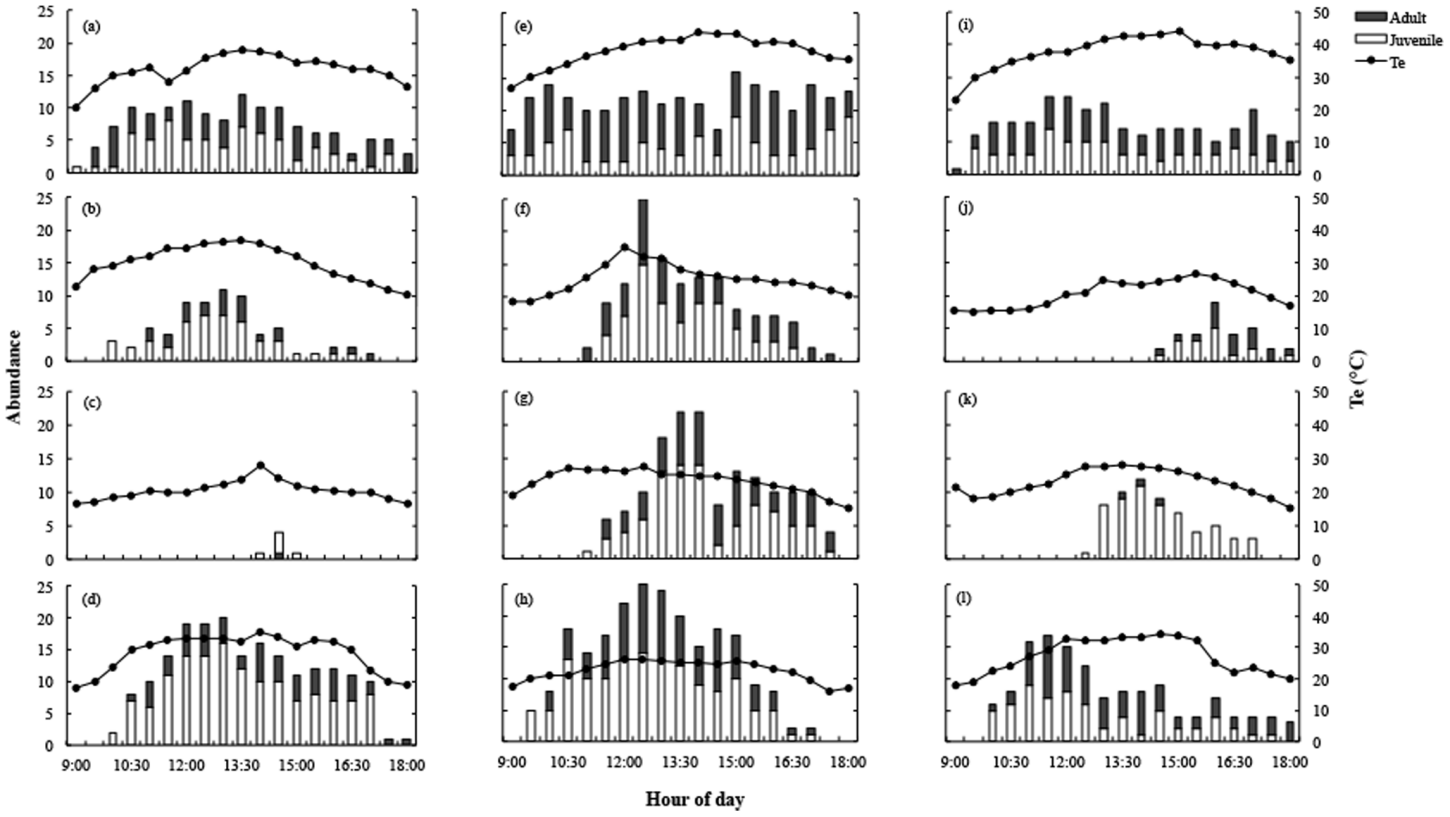
Number of juveniles (white bars) and adults (black bars) *M. atacamensis* lizards along the day in summer, fall, winter, and spring seasons for: Medano (a–d), Zenteno (e–h), and Arrayán (i–l) populations. Circles represent T_e_ (measured with Tidbits data loggers).

Daily activity patterns varied greatly among seasons for the three populations. During summer, lizards emerged from their burrows as early as 0900 h, when T_e_ was above 20°C. Lizards remain active all day, and did not show a clear abundance pattern (Fig. 2). When we finished our observation period (1800 h), we could still observe some individuals above the rocks. Both juveniles and adults emerged at 0900 h, and remained active until 1800 h.

The activity time was shorter in fall and winter, especially for Medano and Arrayán sites. Lizards' appearance coincided with an increase in T_e_ (above 25°C), showing a unimodal activity pattern. In general, juveniles emerged earlier than adults in the three sites and disappear before them in late afternoon. In spring, daily activity pattern was similar to fall and winter, showing a unimodal activity pattern, with maximal abundances near midday for both juveniles and adults. The activity period in spring months was similar to summer, but lizards emerged around 1000 h for the three sites. Again, juveniles emerged earlier than adults, but disappear before them in the afternoon.

**Figure 3:**
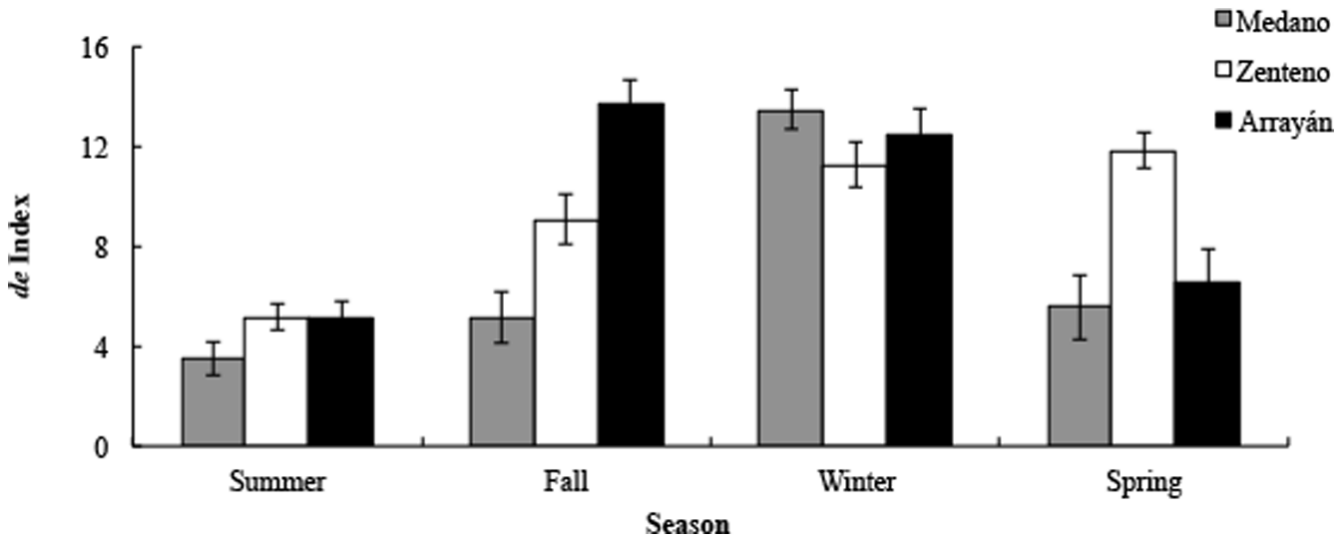
Mean and standard error of the *d_e_* index.

Operative temperature was strongly correlated with the number of observed active lizards, for the three sites studied (τ = 0.62 in Medano; τ = 0.34 in Zenteno; τ = 0.57 in Arrayán) (all Kendall rank correlation coefficients significant at *P*<0.001, seasons pooled). The deviation of T_e_ from T_sel_ (*d_e_*) varied significantly among sites (*F*_2,216_ = 9.19, *P*<0.001), seasons (*F*_3,216_ = 35.52, *P*<0.001), and for the interaction of these two factors (*F*_6,216_ = 8.80, *P*<0.001). Thus in Medano, average *d_e_* was smallest (i.e. a highest thermal quality) than in the other sites. Seasonally, *d_e_* index was lowest in summer and highest in winter. No differences were found between spring and fall (Fig. 3). T_e_ varied considerably with time of day for each season and site. In summer, and for all sites, thermal suitability for lizards was high during the day. In contrast, in the other seasons thermal suitability was lower, especially in early morning and late afternoon, and higher at midday hours, when lizards were active.

**Figure 4:**
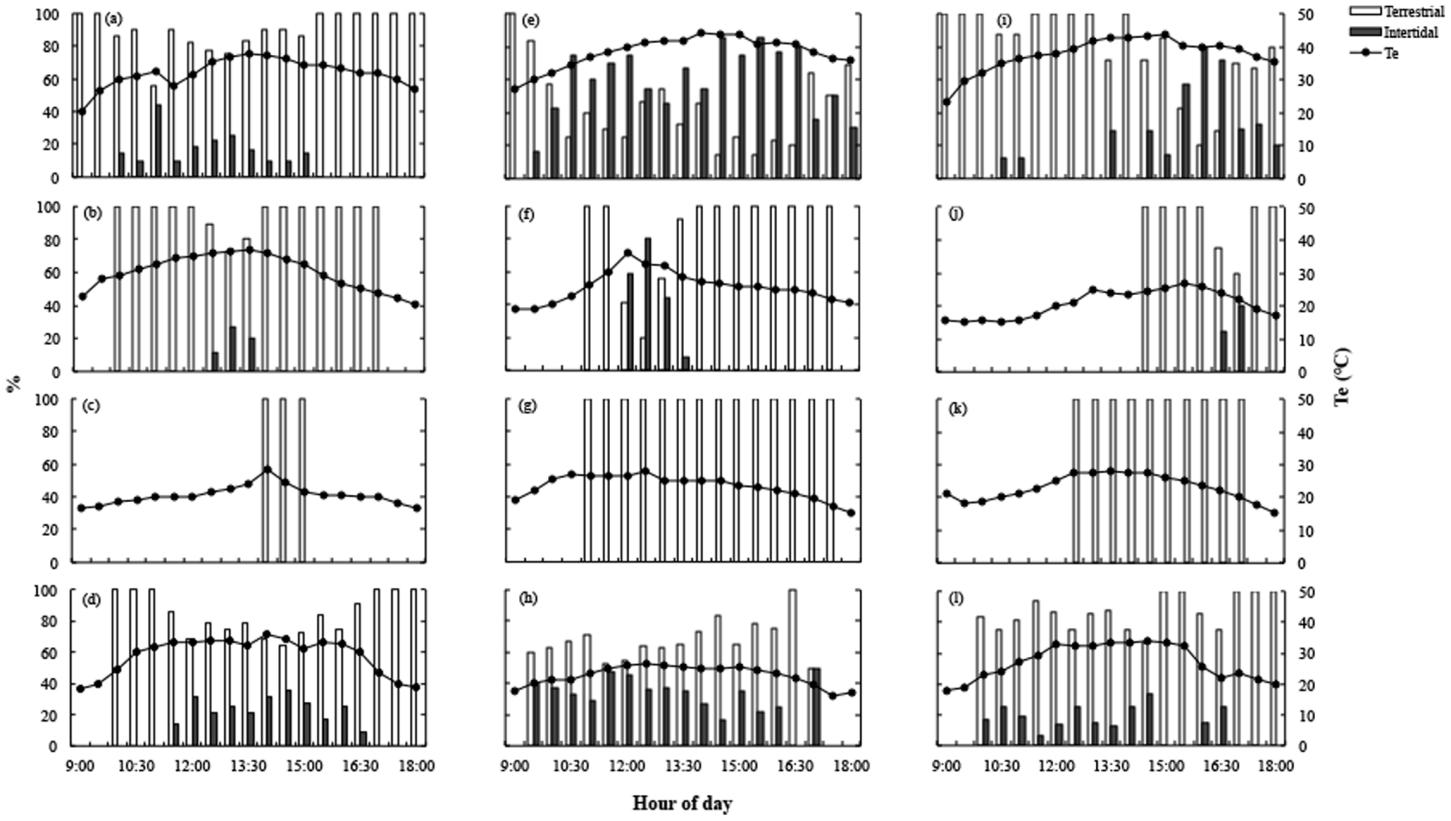
Diel variation of the percentage of active lizards in terrestrial and intertidal habitats in summer, fall, winter, and spring seasons for: Medano (a–d), Zenteno (e–h), and Arrayán (i–l) populations. White bars represent the proportion of individuals in the terrestrial or supralittoral zone; the black ones represent the proportion in the intertidal zone. Maximal abundance in the day was considered as 100%. Circles represent T_e_ (measured with Tidbits data loggers) in the intertidal and terrestrial habitats.

*Microlophus atacamensis* also shows geographical and seasonal differences in the use of intertidal and terrestrial habitats. In Medano, lizards show a clear pattern of use of both habitats. Individuals were found in the intertidal zone when T_e_ surpasses 25°C. In winter, when T_e_ almost never exceeds this temperature, lizards remain in the terrestrial areas (Fig. 4). In summer and spring, lizards from Zenteno and Arrayán were found both in the intertidal and terrestrial habitats during the day. In fall, only a few individuals were found in the intertidal zone during a short period of time. In winter, no individuals were found in the intertidal zone for any site.

### Biophysical simulations

**Figure 5:**
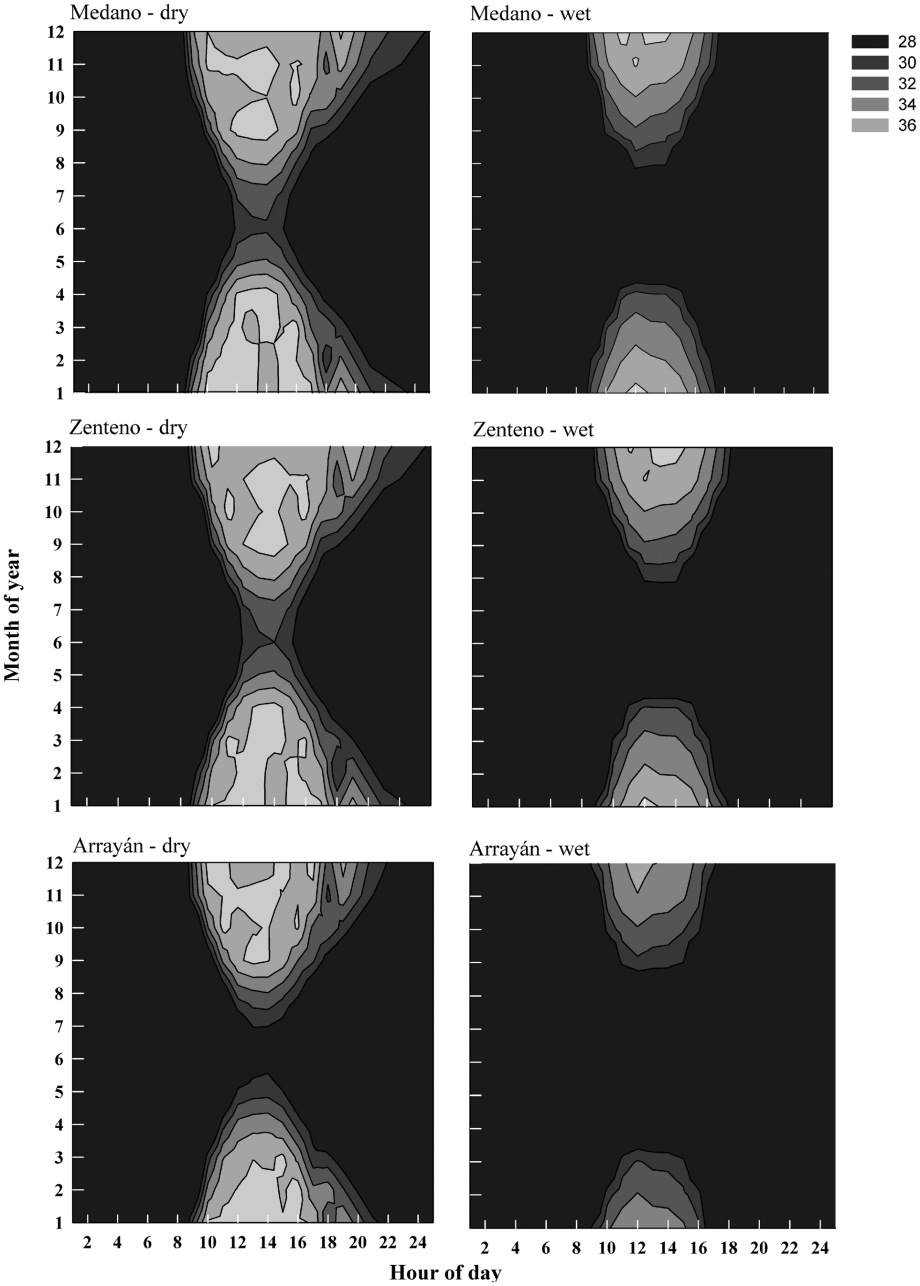
Seasonal and daily activity constraints for *Microlophus atacamensis* in Medano, Zenteno, and Arrayán, on dry (terrestrial) and wet (intertidal) conditions. Gray contours indicate suitable temperatures (28–36°C) for activity.

Calculations of core temperature throughout a typical day in different months and hours of day were made for two different body weights (100 g in males and 36 g in females) in each site, which represent the average weights for both sexes' measured levels of activity. Considering that the voluntary thermal minimum and maximum for lizards are 28 and 37°C, respectively (Table 2), constraints on daily and seasonal potential activity times under the dry (terrestrial) and wet (intertidal) conditions were estimated (Fig. 5). As was observed in the field, our simulations largely reflect seasonal variations in activity times in all sites, with winter activity significantly curtailed by temperature. The thermal constraints on lizard activity precluded the occurrence of *M. atacamensis* in the intertidal habitat in late fall and early winter months, especially for Arrayán site.

**Figure 6:**
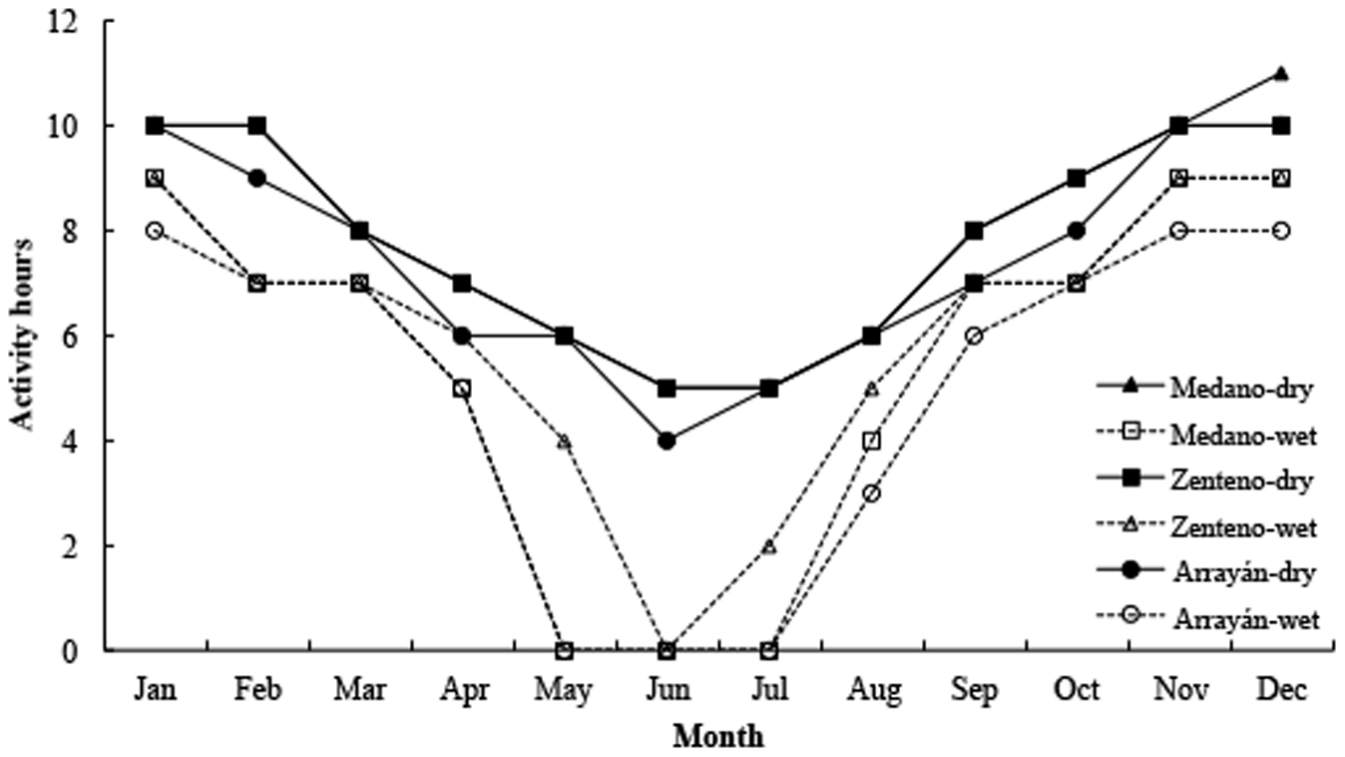
Monthly predicted hours of activity for lizards in Medano, Zenteno, and Arrayán, on dry (terrestrial, filled symbols) and wet (intertidal, empty symbols) conditions.

**Figure 7:**
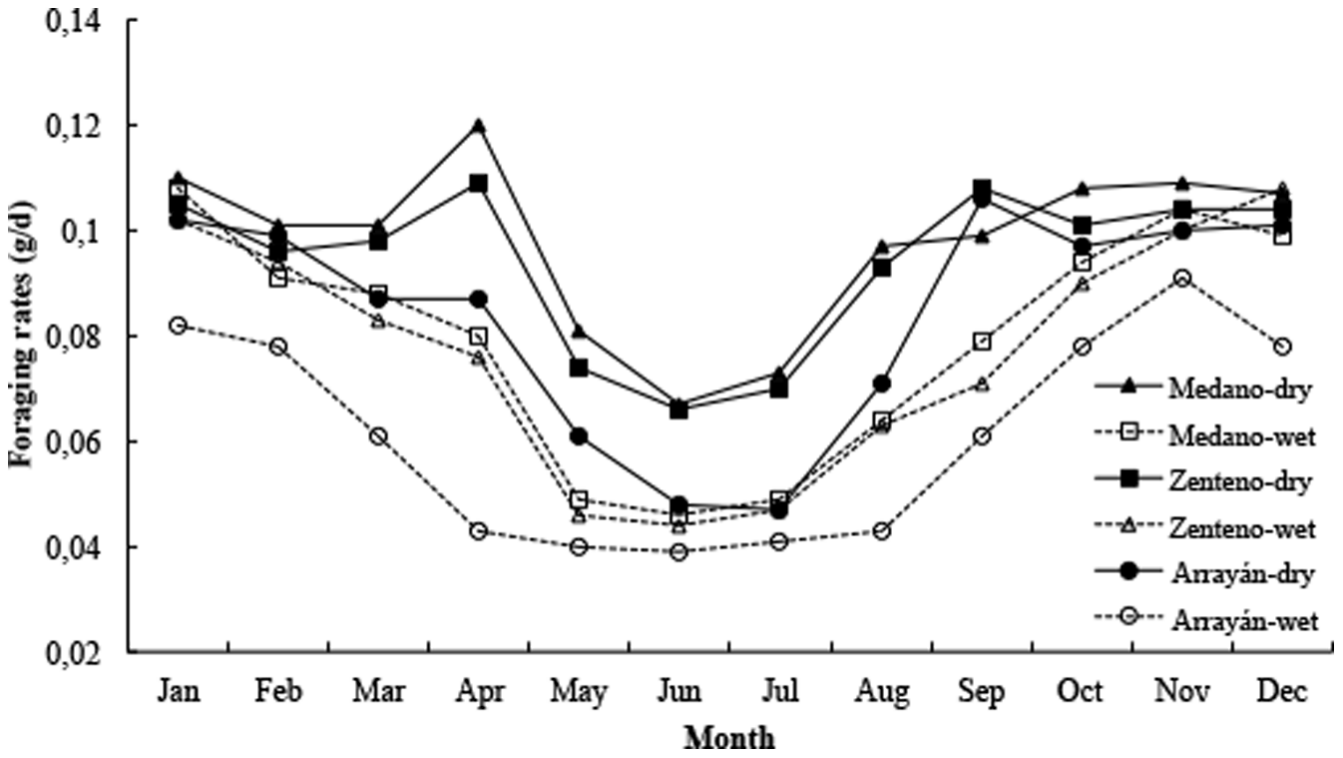
Foraging rates for lizards from Medano, Zenteno, and Arrayán on dry (terrestrial, filled symbols) and wet (intertidal, empty symbols) conditions.

Based on the biophysical model, the activity hours for lizards in the three localities are highest in spring and summer months, when the ability to exploit both habitats is enhanced (Fig. 6). However, the activity decreases in fall and winter months, especially in the intertidal habitat. Potential annual activity time decreases with latitude, ranging from 3099 h yr^−1^ in Medano to 2981 h yr^−1^ in Arrayán in dry conditions, and also in wet conditions from 2918 to 2860 h yr^−1^ in Medano and Arrayán, respectively. Limited activity is manifested in reduced opportunities for feeding, which can be assessed by measuring the required foraging rates in each locality and habitat. In northern sites, and in dry conditions, the foraging rate increases because of increased opportunities for activity (Fig. 7). In wet and southern habitats, lizards are compromised in terms of total annual activity hours, but their annual food requirements are reduced. Through the year, foraging rates are generally higher (∼0.1 g h^−1^) during spring and summer months and lower (<0.07 g h^−1^) during fall and winter seasons.

### Body size

Body size (SVL and mass) differed significantly among sites and sexes (Table 4). For SVL comparisons, females were significantly smaller than males in all the sites studied. However, for each sex, lizards from Medano, Zenteno, and Arrayán were similar in size. With regard to body mass, males were heavier than females. In females, body mass of Arrayán lizards was greater than both Medano and Zenteno lizards. Medano and Zenteno lizards were similar in weight. In the case of males, body mass from Medano lizards was smaller than lizards from Zenteno and Arrayán, but similar between Zenteno and Arrayán.

**Table 4:** ANOVA results for body size comparisons among sites, and sexes.

Dependent variable	Effect	df	MS	F	P-level
SVL	Site	2	0.00	0.9	0.34
	Sex	1	0.21	100.9	**<0.001**
	Site × Sex	2	0.00	1.2	0.31
	Error	118	0.00		
W_b_	Site	2	0.14	6.9	**<0.001**
	Sex	1	2.57	128.8	**<0.001**
	Site × Sex	2	0.03	1.7	0.19
	Error	126	0.02		

SVL: Snout-vent length, W_b_: body mass.

## Discussion

Our results indicate that the latitude and climate are important factors in modulating the activity patterns of *M. atacamensis* lizards. We predicted that lizards from northern sites should have longer daily and seasonal activity times because they experience a warmer environment. Accordingly, we found a small *d_e_* and a higher potential annual activity time in Medano, implying that this northern locality offers better quality thermal conditions for the activity of lizards [13]. A decrease in potential activity time in the southernmost site (Arrayán) is explained because the thermal quality of the environment in this locality is lower. It is likely that lizards from this site compensate for the high time cost of thermoregulation by allocating more time to basking than to other activities [31], which in turn may imply a trade-off between feeding and thermoregulation. An increase in basking time activities can affect foraging time and consequently the energy budget [32], and ultimately the reproductive success of lizards in high-latitude populations. The abundance of lizards in Arrayán was significantly lower than in Medano and Zenteno, suggesting that in this southern locality the animals were under a higher cost of thermoregulation. Lizards from Arrayán should allocate more time to thermoregulation with a consequently smaller proportion of the time budget allocated to foraging, thus decreasing the energy available for growth and reproduction. It is important to note that the smaller population size in Arrayán compared to Medano and Zentano might be also due to higher predation (e.g. more birds and snakes in a more mesic environment) on adult and juvenile lizards [18].

We found that these lizards also shift their daily activity times during the year. In summer and spring the daily activity time was longer, and reduced in the fall and winter seasons as expected for diurnal lizards [6], [33], [34]. Our biophysical model also revealed similar results, with a longer period of activity in summer and spring and lower in fall and winter, providing good correspondence with empirical data. Similar to the differences among localities, longer daily activity times found in summer and spring could be explained because the *d_e_* index was lower in these seasons, indicating a high thermal quality for the activity of lizards.

During the fall, winter and spring seasons, lizards showed a unimodal activity pattern, being active around midday when solar radiation and T_e_ were high. This pattern is typical of several species of lizards [35], [36]. In particular, *M. peruvianus* (a species that belongs to the *M. peruvianus* group, as does *M. atacamensis*) also shows a unimodal pattern of activity during this period of time [20]. Nevertheless, a shift in activity from unimodal to bimodal has been found in several species of diurnal desert lizards in summer [6], [37], [38], generally observed at sites where air and soil temperatures reached risky highest levels. Around midday, lizards retreat into the shade or burrow to avoid overheating, but become active again during the afternoon and thus exhibit a markedly bimodal activity pattern [37]. Traditionally, this daily pattern of activity has been explained as the behavioral response of these animals to the very high levels of air and soil temperatures at that time of day [39]. However, laboratory experiments in some species of lizards demonstrate that summer bimodality is not merely a proximate response of lizards to high levels of soil temperature, but is a clock driven phenomenon mediated by circadian rhythmic changes of plasma melatonin levels [40], [41], [42], which has most probably evolved as a behavioral adaptation to high levels of air temperature occurring predictably around midday [5], [43].

Regardless of the mechanism associated with a unimodal/bimodal activity pattern, during summer *M. atacamensis* faces a stressful heat load in midday hours, which should prohibit activity during these hours. However, they did not show a bimodal pattern of activity, but interestingly they used the behavioral strategy of moving to the intertidal habitat, where temperatures are lower and wind speed on the shore is greater. This strategy of lizards of moving to the intertidal habitat as the temperature rises allows them to engage in continuous daily activity associated with the use of different substrates, extending their activity period close to a source of food, maintaining a similar temperature during the day [44]. Also, this behavior benefits lizards because food and energy requirements in wet habitats are reduced. However, it also represents costs for thermoregulation, because the low substrate and air temperatures in the intertidal habitat increase the heat lost by conduction, substrate radiation and local air temperature due to local substrate evaporation [20].

Body temperature showed significant variation throughout the year, being lower in fall and winter months [21]. Biophysical simulations using Niche Mapper also demonstrated that food intake and foraging rate were lower during these seasons. Lower T_b_ in winter compared to summer suggests that in winter lizards are under environmental constraints for thermoregulation and should operate as low-energy systems [20], [21]. Thus there appears to be a trade-off in choosing the use of terrestrial and/or intertidal habitats by a lizard. In summer when T_e_ in the intertidal habitat is >25°C lizards use this microhabitat, extending their daily activity time and accepting the physiological cost of heat loss (i.e. a decrease in metabolic rate), while in seasons other than summer, the cost of heat loss appears to surpass the benefits and lizards are restricted in their visits to the intertidal habitat. Thus the net ecological benefit establishes the use of different microhabitats and consequently the activity period at seasonal (and geographical) scales.

It is interesting to note that, although northern locations and dry conditions offer a considerable advantage in terms of time spent within optimal limits [44], the access of lizards to wet habitats give them the advantage of more food, reducing their energy requirements. For these reasons, spatial and temporal differences in the access to the intertidal habitat could affect energy acquisition differentially for populations of *M. atacamensis*. A latitudinal variation in the diet composition of *M. atacamensis* was found, with an increase in the proportion of terrestrial items in a north to south gradient [18]. The diet of lizards from Medano (northernmost site) is composed almost exclusively of marine invertebrates and algae, thus lizards from this locality may be under a trade-off between feeding and thermoregulation, which may in turn determine differences in the use of the thermal substrate. Due to the lower T_e_, except in the warmest months, lizards from Medano are restricted in visits to intertidal zones and this would reduce the ingestion of intertidal prey. This energy constraint might explain the lower body mass found in Medano lizards. Although longer seasonal activity is usually associated with larger body size [45], we found that lizards (both males and females) from Arrayán, and not from Medano, were larger.

However, there are other possible contributing factors for the lack of relationship between activity duration and body size which should be considered. Together with a variation in temperature along the gradient there is a consistent increase in rainfall to the south, which promotes productivity. Lower abundance of terrestrial invertebrates available for lizards may force northern lizards to exploit intertidal feeding because of the lack of insects in the extremely dry supralittoral zone. Thus even with more activity time they may be food limited, which could either stunt growth resulting in smaller body size or accelerate sexual maturation [46], [47], which could also account for the observed smaller body sizes. Another hypothesis is that longer times at higher body temperature lead to higher metabolic rates and therefore more food and water are needed to sustain those higher rates [48]. In fact, Maritza Sepúlveda (unpublished data) found that lizards from Medano exhibited higher metabolic rates than lizards from Zenteno and Arrayán. If food and/or water are limited, greater metabolic expenditures in the face of limited food could also affect the potential growth. Finally, due to the lower temperature and a shorter activity season, lizards from Arrayán may need to extend the time of sexual development, resulting in larger body size at sexual maturity [13].

The activity pattern of *M. atacamensis* differs between juveniles and adults. We found that juveniles emerge before adults and become inactive earlier in the afternoon. At least three not mutually exclusive explanations can be hypothesized. First, because smaller lizards heat up quickly and larger lizards cool down slowly [6], [21], it has been suggested that body size should play an important role in determining the activity pattern of large versus small lizards [49]. Such an effect has been argued to explain why during the day juveniles of desert reptiles are active earlier and adults later [50]. Second, it is possible that intraspecific social interactions lead to a temporal segregation between age classes. In this vein, a highly territorial behavior of adults and aggressive behavior towards juveniles was found [23], so it is possible that juveniles emerge earlier to minimize interactions with adults. Finally, some preliminary data suggest that juveniles and adults may be exploiting different classes of prey. José Miguel Fariña (unpublished data) found a relatively high proportion of Diptera in the diet of juvenile lizards compared to the diet of adults, which suggests that there may be a lack of overlap in food resources.

In conclusion, we found that the thermal environment is a key factor that modulates the daily and seasonal activity of *M. atacamensis* lizards, influencing their abundance, patterns of activity, foraging rates and body size. Lack of some thermal physiological adjustments among populations of this species [21] indicates that *M. atacamensis* mostly relies on behavioral strategies to cope with spatial and temporal variation in the thermal environment. The cost/benefit relationship appears to be critical in the use of the intertidal habitat by a lizard, representing a trade-off between thermoregulation and feeding. While the use of intertidal habitat does not offer high quality thermal conditions, it allows them to extend behaviorally their activity window and gain access to food in the intertidal areas. Because its use of intertidal habitat means that this species is not required to display a bimodal pattern, it will be interesting to test whether or not this species exhibits a bimodal pattern controlled by endogenous temporal programs under stressful thermal conditions. This kind of information will add to our understanding of the underlying evolutionary causes of the lizard's circadian system.
